# Targeting CD39 as a Therapeutic for Cancer Immunotherapy

**DOI:** 10.1017/erm.2026.10033

**Published:** 2026-01-26

**Authors:** Zhongliang Li, Weiguo Xu, Hang Fai Kwok

**Affiliations:** 1 Department of Biomedical Sciences, Faculty of Health Sciences, University of Macau, Avenida da Universidade, Taipa, Macao and Zhuhai People’s Hospital Centre for Precision Medicine, Zhuhai Clinical Medical College of Jinan University, Zhuhai, Guangdong, China; 2 Department of Cerebrovascular Disease, Center of Intervention Radiology, Zhuhai People’s Hospital (The Affiliated Hospital of Beijing Institute of Technology, Zhuhai Clinical Medical College of Jinan University), Zhuhai, Guangdong, China; 3 Department of Biomedical Sciences, Faculty of Health Sciences, University of Macau, Avenida de Universidade, Taipa, Macao

**Keywords:** CD39, immunotherapy, immune evasion, immune suppression, tumour microenvironment

## Abstract

CD39 plays a pivotal role in the ATP-to-adenosine signalling pathway, serving as a critical mediator of immune suppression within the tumour microenvironment. Increasing preclinical evidence indicates that its inhibition can restore antitumour immunity and improve the efficacy of established treatments. In this review, we summarise the biology of CD39, its role in shaping the immunosuppressive tumour microenvironment, and therapeutic strategies currently under development. We also discuss early clinical progress and safety considerations, along with major challenges and future perspectives. Targeting CD39 represents a promising strategy to overcome tumour-induced immunosuppression and ongoing advances in therapeutic development could usher in next-generation immunotherapies.

## Introduction

Cancer immunotherapy has revolutionised the treatment of malignancies by utilising the immune system to selectively recognise and eliminate cancer cells, thereby restraining tumour progression and enhancing clinical outcomes (Ref. 1). The immune checkpoint inhibitors (ICIs), which are monoclonal antibodies against the programmed death-1/programmed death-ligand 1 (PD-1/PD-L1) axis and cytotoxic T-lymphocyte–associated protein 4 (CTLA-4), have shown significant clinical benefits in various cancer types (Refs 2, 3). Yet, many patients fail to respond or become resistant over time, indicating the urgent need for novel strategies that overcome immune escape and broaden the benefits of immunotherapy (Ref. 4).

Among emerging metabolic checkpoints, CD39 (ectonucleoside triphosphate diphosphohydrolase-1, ENTPD1) has attracted increasing attention for its pivotal role in shaping the immunosuppressive tumour microenvironment (TME) (Refs 5, 6, 7, 8, 9, 10). By reinforcing immune evasion, elevated CD39 expression correlates with poor prognosis in multiple cancers, including ovarian, renal, breast and gastric cancers (Refs 11, 12, 13, 14). Preclinical studies have revealed that targeting CD39 can stimulate T cell activation, alleviate regulatory T cell (Treg)-mediated suppression, and improve the effectiveness of immune checkpoint blockade therapies (Refs 10, 15, 16, 17). However, several translational challenges remain. The broad physiological expression of CD39, particularly on endothelial cells and platelets, raises safety concerns; redundancy within the adenosinergic pathway may attenuate therapeutic efficacy and heterogeneity in spatial and cellular expression complicates patient selection in clinical practice (Refs 18, 19, 20, 21, 22). Despite these barriers, multiple anti-CD39 monoclonal antibodies have now advanced into early-phase clinical trials, providing the early evidence of safety and target engagement in patients.

This review integrates mechanistic and translational insights on CD39, with particular attention to its dual role in immune regulation and vascular biology, spatial heterogeneity as a biomarker and ongoing efforts in therapeutic development. Clarifying these aspects is essential to defining the opportunities and challenges of CD39 blockade as part of next-generation cancer immunotherapy.

## Biology and mechanisms of CD39

### Structure and enzymatic function of CD39

CD39 functions as a vital transmembrane enzyme that belongs to the ectonucleotidase family to perform cell surface nucleotide hydrolysis (Ref. 23). The CD39 protein contains two transmembrane domains, intracellular N- and C-termini and a large extracellular loop containing five apyrase-conserved regions (ACR1–ACR5), among which ACR1 and ACR4 are most critical for ATP/ADP hydrolysis (Refs 24, 25). CD39 is anchored to the cell membrane by the transmembrane domains. The ATP and ADP hydrolysis takes place at the active site positioned in the extracellular large loop, while the ACRs enable the enzymatic function of CD39. The enzymatic reaction that occurs through CD39 needs divalent cations such as Ca^2+^ and Mg^2+^ to stabilise the substrate-binding site and enhance the conversion of ATP and ADP into AMP (Ref. 26). Through this activity, CD39 regulates extracellular nucleotide concentrations by reducing pro-inflammatory ATP/ADP signals and generating AMP, the substrate for CD73-mediated adenosine production.

### The adenosinergic signalling pathway

**Figure 1.**
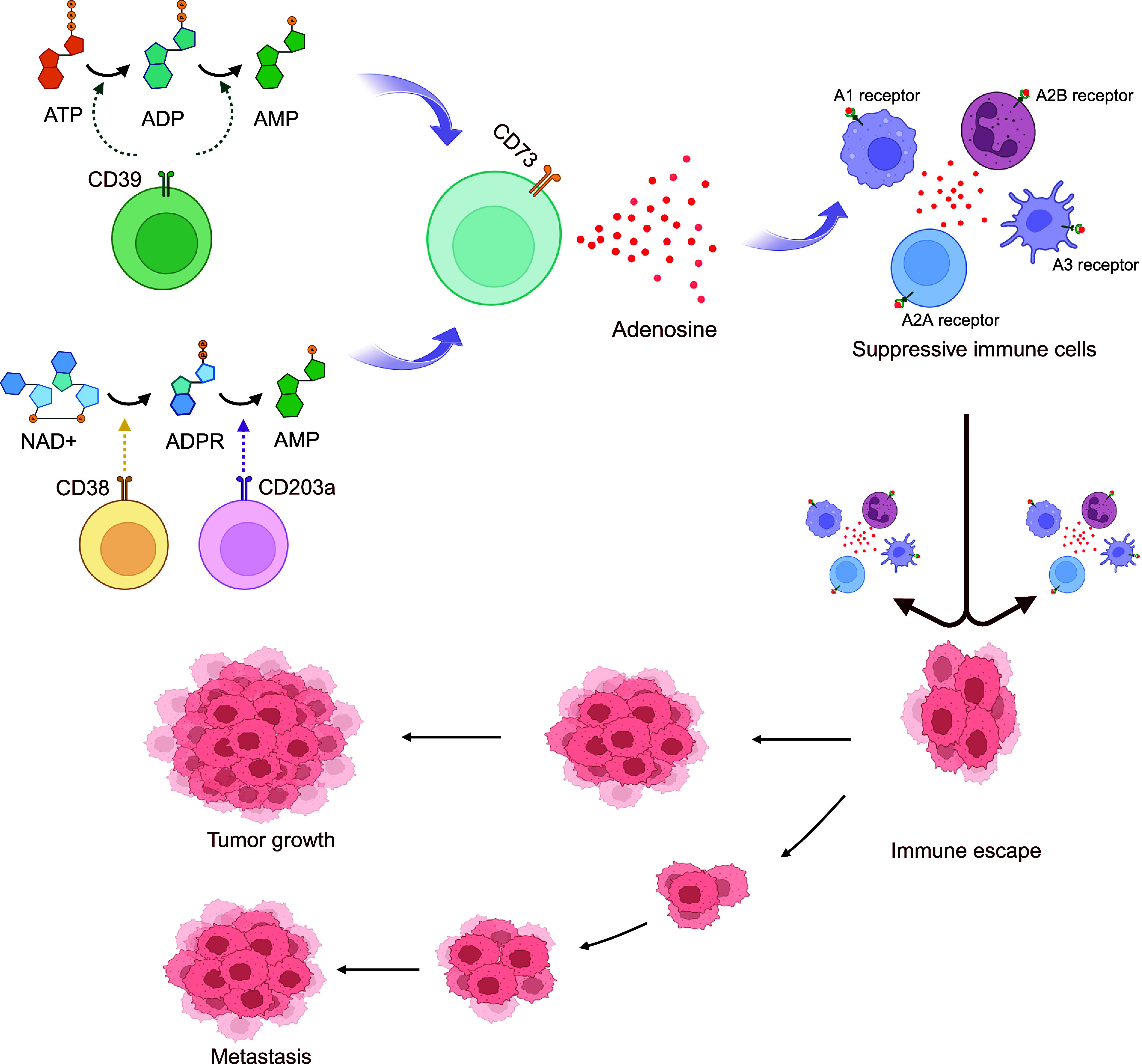
The adenosinergic signalling pathway.

The adenosinergic signalling pathway, with CD39 as its dominant regulator, encompasses both canonical and non-canonical routes that collectively sustain immune suppression within the TME (Ref. 7). In the canonical pathway, CD39 hydrolyses extracellular ATP and ADP into AMP, which is subsequently converted into adenosine by CD73 (Figure 1) (Refs 8, 24, 27, 28). Non-canonical pathways also contribute, including the CD38–CD203a axis that degrades NAD^+^ into ADPR and then into AMP, which is also converted into adenosine by CD73 (Figure 1) (Ref. 29). Adenosine then interacts with specific purinergic receptors (A1, A2A, A2B and A3), profoundly impacting the function of immune cells (Figure 1) (Refs 7, 8, 30). When adenosine binds to the A2A receptor on cytotoxic T lymphocytes (CTLs) and natural killer (NK) cells, it substantially reduces their cytotoxic activity, impairing their ability to identify and destroy tumour cells. In addition, adenosine also enhances the proliferation and suppressive activity of Tregs, disrupts dendritic cell (DC) maturation and antigen presentation and augments the immunosuppressive phenotype of myeloid-derived suppressor cells (MDSCs) (Refs 7, 30, 31, 32, 33). Together, through this pathway, CD39 and CD73 create a tumour-supportive, immunosuppressive microenvironment that facilitates tumour growth, metastasis and resistance to therapies (Ref. 34).

Beyond immune regulation, CD39 plays essential roles in vascular biology. On endothelial cells and platelets, it rapidly hydrolyses ATP/ADP to inhibit platelet aggregation and prevent microvascular thrombosis, thereby maintaining vascular integrity (Refs 35, 36). This dual role as both an immune checkpoint and a thromboregulatory enzyme creates a therapeutic paradox, in which systemic CD39 blockade may reinvigorate antitumour immunity but simultaneously destabilise vascular homeostasis if not carefully monitored.

## CD39 and cancer

### CD39 expression across different cell types

CD39 expression is widely detected across malignant, stromal, and immune compartments of tumours. High levels have been consistently reported on tumour-infiltrating lymphocytes, particularly Tregs, MDSCs, tumour-associated macrophages (TAMs) and exhausted CD8^+^ T cells (Refs 12, 33, 37, 38, 39). In melanoma, CD39 is enriched on both tumour cells and Tregs (Refs 40, 41), whereas in colorectal cancer (CRC) and non-small-cell lung cancer (NSCLC), it is prominent on tumour-infiltrating lymphocytes (TILs) (Refs 5, 41, 42, 43). Similarly, the expression of CD39 reaches high levels in ovarian cancer tissue, where it also appears on Tregs and MDSCs (Ref. 13). Beyond immune subsets, stromal cells such as cancer-associated fibroblasts (CAFs) and endothelial cells express CD39, linking adenosine metabolism to angiogenesis and vascular remodeling (Refs 44, 45). Platelets extend this regulation systemically, with platelet-expressed CD39 modulating thrombosis and circulating nucleotide balance (Refs 45, 46). These findings establish CD39 as a central regulator across diverse cellular compartments, which makes it a promising therapeutic target.

### Prognostic significance of CD39 in cancer

Multiple studies demonstrate that CD39 functions as a marker of poor prognosis across various cancers, including melanoma, colorectal and ovarian cancers (Refs 13, 40, 42). In melanoma, the elevated CD39 expression on tumour cells and Tregs creates an immunosuppressive microenvironment that makes PD-1/PD-L1 blockade therapies less effective (Ref. 40). Similarly, in CRC and NSCLC, CD39 expression is highly prominent on TILs and correlated with a poor prognosis, lower response rates to immunotherapy and reduced overall survival (Refs 42, 43). Notably, in prostate adenocarcinoma, CD39^+^ Tregs have been shown to promote epithelial–mesenchymal transition (EMT) through adenosine signalling, highlighting a distinct mechanism by which CD39 contributes to tumour progression (Refs 34, 47). In addition, ovarian cancer is characterised by CD39^+^ TAMs that enhance adenosine production, thereby amplifying immune suppression and supporting tumour progression (Ref. 48). Likewise, in pancreatic cancer, CD39 expression on TAMs is associated with elevated adenosine levels, driving tumour progression and inhibiting effective antitumour immunity (Ref. 49). These clinical observations suggest CD39 is a marker of immune suppression and a crucial contributor to tumour progression, holding a substantial potential as a valuable prognostic biomarker.

### Roles of CD39 in the TME

**Figure 2.**
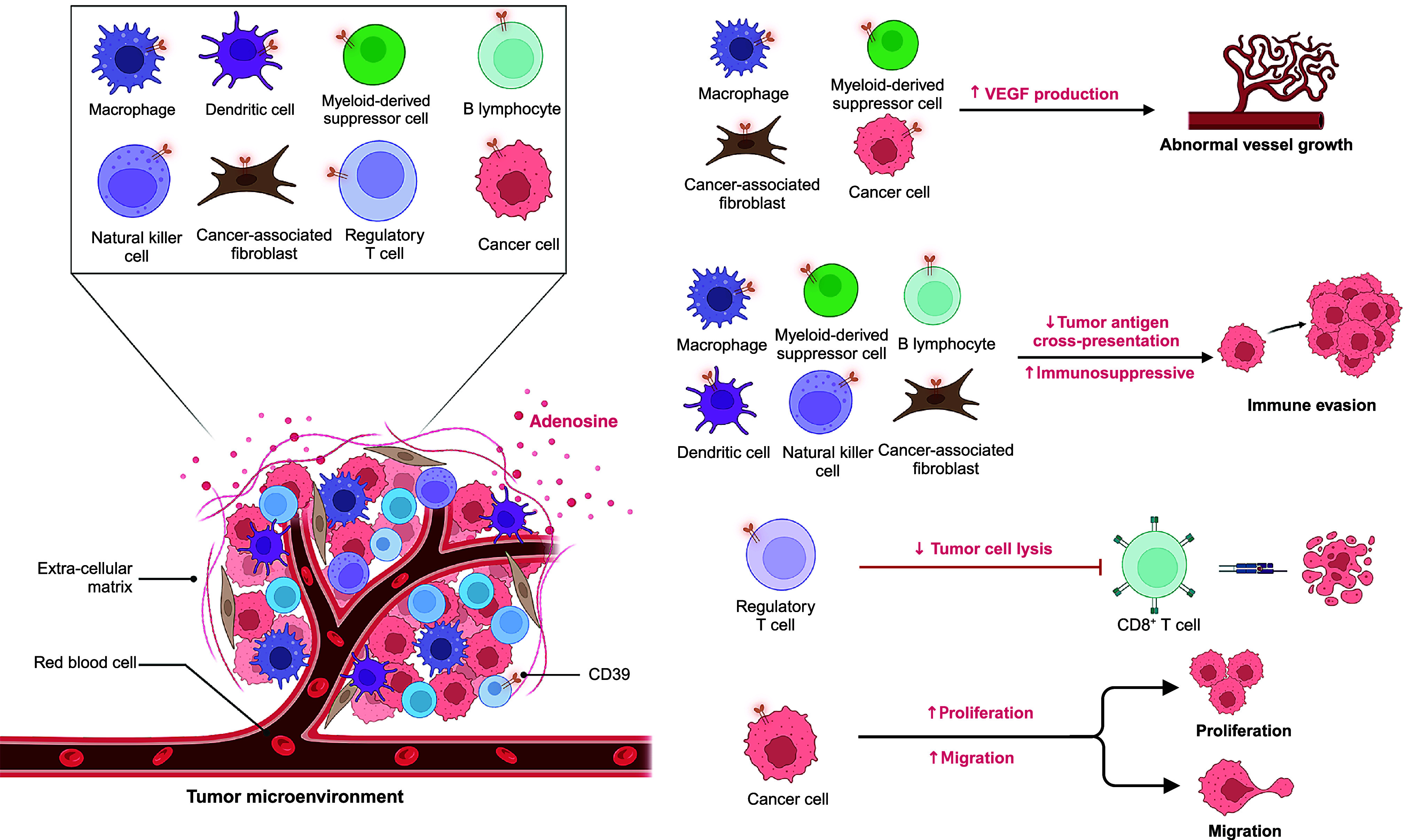
Roles of CD39 in the TME.

Within tumour, stromal and immune compartments, CD39 constrains immune surveillance predominantly via adenosine-mediated suppression, with CAFs and endothelium further reinforcing this environment (Refs 50, 51, 52, 53, 54, 55). On tumour cells, CD39 directly contributes to immune escape by degrading extracellular ATP, thereby reducing pro-inflammatory danger signals and enabling the accumulation of immunosuppressive adenosine (Refs 56, 57). The impact of CD39 expression extends beyond the tumour cells themselves. In the stromal compartment, CAFs and endothelial cells express CD39, linking adenosine metabolism to tumour angiogenesis, vascular remodelling and metastatic spread (Refs 11, 34, 57). CD39 is also highly expressed in the immune component of the TME. Within the immune infiltrate, CD39 is enriched on Tregs, MDSCs, and TAMs, where it synergises with CD73 to generate adenosine, suppress CTLs and NK cells and amplify tolerogenic circuits (Figure 2) (Refs 48, 50, 58, 59). Beyond its immunoregulatory role, CD39 also shapes tumour biology through control of extracellular ATP. While high ATP levels can promote proliferation and migration via P2X/P2Y receptor signalling, CD39 hydrolysis dampens these effects but simultaneously drives adenosine-A2B receptor signalling, which enhances angiogenesis and metastasis (Refs 33, 47, 60, 61, 62, 63, 64). Recent spatial studies further underscore the multifaceted biology of CD39, showing that it cooperates with CD73 in hypoxic niches to drive adenosine-mediated immunosuppression in glioblastoma, while also marking tumour-reactive CD8^+^ T cells with exhaustion gradients at invasive margins in esophageal cancer. (Refs 22, 65). These spatial insights position CD39 not only as a mediator of suppression but also as a spatial biomarker with potential utility for patient stratification and trial enrichment.

## Therapeutic strategies targeting CD39

**Figure 3.**
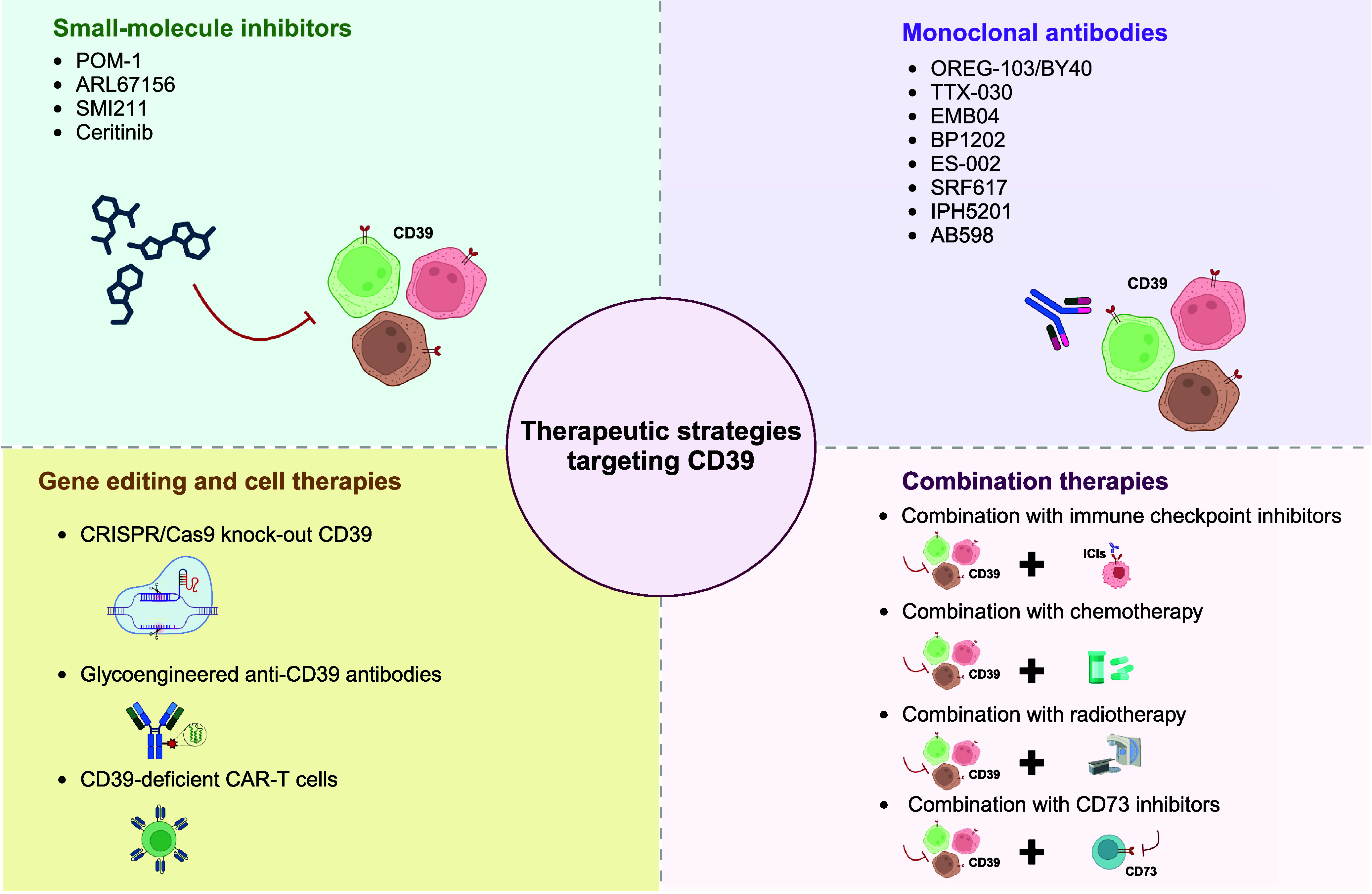
Therapeutic strategies targeting CD39.

Given its role in modulating immune suppression, CD39 has emerged as a promising target for therapeutic strategies to reinstate antitumour immunity and enhance the effectiveness of existing immunotherapies (Refs 10, 66). Current strategies to target CD39 include small-molecule inhibitors, monoclonal antibodies, gene editing-based cell therapies and combination treatment approaches (Figure 3) (Ref. 6).

### Small-molecule inhibitors

Small-molecule inhibitors represent a primary method to directly block CD39 enzymatic activity (Table 1). These compounds function through ATP conversion inhibition, which leads to the reduction of immunosuppressive adenosine in the TME. By reversing the immunosuppressive conditions, these agents can boost T cell activation, enhance CTL function, and promote T cell infiltration into the tumour, ultimately strengthening antitumour immunity (Ref. 67).

**Table 1.** Preclinical antagonists targeting CD39

Name	Chemical class	Mainly effects	References
POM–1 (sodium polyoxotungstate)	Small-molecule inhibitor	Targets both CD39 and CD73, reduces adenosine accumulation	(Refs 40, 68)
ARL67156	Small-molecule inhibitor	Prevents ATP hydrolysis	(Refs 69, 70, 71)
SMI211	Small-molecule inhibitor	Reduces AMP production	(Ref. 16)
Ceritinib	Small-molecule inhibitor	Reducing adenosine production	(Ref. 72)
OREG–103/BY40	Monoclonal antibody	Reduces adenosine buildup in the TME	(Ref. 40)
9–8B	Monoclonal antibody	Blocked CD39-mediated ATP hydrolysis	(Ref. 80)
TTX–030 (Trishula)	Monoclonal antibody	Restoring T cell activation and cytotoxicity	(Refs 19, 73, 74)
EMB04	Monoclonal antibody	Preventing adenosine buildup	(Ref. 82)
BP1202	Monoclonal antibody	Enhances T cell functions	(Ref. 81)
ES–002	Monoclonal antibody	Restoring immune-mediated tumour control	(Ref. 83)
SRF617	Monoclonal antibody	Enhances T cell activation	(Refs 75, 76)
IPH5201	Monoclonal antibody	Enhances ATP-driven immune responses	(Refs 77, 78)
AB598	Monoclonal antibody	Restore antitumour immune responses	(Ref. 79)

Several compounds have shown preclinical efficacy. POM-1 (sodium polyoxotungstate) is a broad-spectrum ectonucleotidase inhibitor that blocks both CD39 and CD73, reducing adenosine levels and improving T cell infiltration in melanoma models (Refs 40, 68). ARL67156, a competitive CD39 inhibitor, prevents ATP hydrolysis and has been reported to boost pro-inflammatory immune responses in colorectal and breast cancer models (Refs 40, 69, 70, 71). More selective agents are under investigation, including SMI211, identified by high-throughput screening, which markedly reduces AMP production in colorectal cancer cell lines (Ref. 16). Interestingly, ceritinib, a clinically approved ALK tyrosine kinase inhibitor, has also demonstrated off-target inhibition of CD39, suggesting a dual mechanism that combines oncogenic pathway blockade with modulation of the immunosuppressive TME (Ref. 72).

### Monoclonal antibodies

Monoclonal antibodies (mAbs) targeting CD39 have garnered significant interest in cancer immunotherapy due to their high specificity, extended half-life and ability to effectively inhibit both the enzymatic activity and expression of CD39 (Table 1). By targeting CD39, these mAbs disrupt the adenosinergic pathway, reducing the immunosuppressive effects of adenosine within the TME.

Several promising anti-CD39 mAbs are being developed, showing strong preclinical efficacy, with some progressing to clinical trials.

TTX-030 (Trishula Therapeutics), the most advanced candidate, is a fully humanised IgG1 antibody that inhibits CD39 enzymatic activity without depleting CD39-expressing cells (Ref. 73). Early-phase trials are evaluating TTX-030 in combination with PD-1 blockade or chemotherapy for advanced solid tumours, with preliminary evidence of restored T-cell cytotoxicity and improved patient responses (Refs 19, 74). IPH5201 and SRF617 are also in clinical trials, where they are being investigated alongside ICIs to enhance effector T-cell infiltration and overcome adenosine-driven resistance (Refs 75, 76, 77, 78). AB598 is undergoing evaluation with chemotherapy and immunotherapy, highlighting the potential of CD39 blockade to counteract treatment-induced immunosuppression (Ref. 79). Beyond these clinical candidates, several other antibodies remain at the preclinical stage, including OREG-103/BY40, 9-8B, BP1202, EMB04 and ES-002, which have shown efficacy in models of melanoma, colorectal cancer and multiple myeloma (Refs 40, 80, 81, 82, 83). These findings demonstrate proof-of-concept that antibody-mediated CD39 inhibition can reverse immunosuppression across both solid and hematologic malignancies (Ref. 28). As clinical trials progress, these therapies hold the potential to revolutionize the treatment of solid tumours and hematological cancers, offering renewed hope for patients with hard-to-treat malignancies.

### Gene editing and cell therapies

Gene-editing technologies, particularly CRISPR/Cas9, have become potent tools for directly targeting CD39 by silencing its expression in immune or tumour cells. In T cells, CD39 deletion leads to greater ATP-dependent T cell activation that increases their effector functions and opposes adenosine-dependent immune suppression in the TME, which enable them to properly eliminate cancer cells, especially in solid tumour models. Similarly, deletion of CD39 from tumour cells creates an immune-permissive environment, leading cancer cells to become more sensitive to immune destruction and overcoming immune evasion in preclinical cancer models (Refs 84, 85, 86). Building on this concept, engineered chimeric antigen receptor T (CAR-T) cells lacking CD39 have demonstrated superior persistence, cytotoxicity, and resistance to adenosine-driven exhaustion, particularly in solid tumour settings where immune suppression is most pronounced (Refs 33, 87).

In addition to gene editing strategies, glycoengineered anti-CD39 antibodies represent an innovative approach. These antibodies bind to CD39 for enzymatic inhibition, which stops the TME from accumulating the immunosuppressive agent adenosine. In the colon cancer model, when combined with ICIs, the glycoengineered anti-CD39 antibodies can remove Tregs and MDSCs while blocking angiogenesis to stop tumour progression and metastasis, demonstrating their potential for cancer therapy (Ref. 88).

Given the great promise of these gene editing-based strategies for cancer therapy, a combination of these strategies with checkpoint inhibitors or metabolic modulators might offer a powerful solution to neutralise immune suppression within the TME.

### Combination therapies

Given CD39’s central role in establishing an immunosuppressive TME, monotherapies are unlikely to achieve durable benefit. Rational combination strategies are therefore emerging as the most promising approach to restore antitumour immunity and overcome resistance to existing treatments. Preclinical and early clinical studies have demonstrated that CD39 blockade synergises with ICIs, chemotherapy, radiotherapy and CD73 inhibition (Refs 33, 89, 90).

One of the most promising approaches for CD39 inhibition is its combination with ICIs. By relieving adenosine-mediated suppression of T cells, CD39 inhibitors enhance checkpoint efficacy, reinvigorate exhausted T cells and promote tumour regression (Ref. 33). Preclinical models of liver and colorectal cancer have confirmed this synergy, and early-phase trials with agents such as TTX-030 and IPH5201 are showing encouraging signs of improved responses in solid tumours (Refs 19, 77).

CD39 inhibition has also demonstrated synergistic effects when combined with chemotherapy and radiotherapy, both of which are known to induce immunogenic cell death (ICD) (Ref. 33). However, the immunosuppressive TME restricts these treatments from achieving their full potential. CD39 inhibition could enhance the immunogenic response by preserving extracellular ATP levels while allowing immune cells to enter the tumour and resulting in a strengthened immune response (Ref. 90). For instance, combining TTX-030 with chemotherapy in pancreatic ductal adenocarcinoma has shown encouraging activity, while pairing IPH5201 with oxaliplatin enhanced ATP-mediated immune responses and improved antitumour efficacy (Refs 15, 74, 77, 78).

Another promising approach is dual inhibition of CD39 and CD73, which act sequentially in the adenosinergic cascade. CD39 generates AMP, and CD73 converts it into adenosine; targeting both enzymes prevents adenosine buildup more comprehensively than CD39 inhibition alone (Ref. 91). Preclinical studies have demonstrated that combined blockade restores T-cell function, drives tumour regression and enhances the activity of ICIs and CAR-T therapies (Refs 15, 92). This strategy is increasingly viewed as a rational approach to disrupt the adenosine pathway at multiple levels.

## Clinical development of CD39-targeted therapies

Since 2020, multiple CD39-targeted agents have transitioned from preclinical discovery into early-phase clinical trials across solid and haematologic malignancies.

TTX-030, the most clinically advanced antibody, has been tested in PDAC and gastric cancer. In the first-in-human trial (NCT03884556), TTX-030 was well tolerated, with no dose-limiting toxicities observed across single-agent cohorts. Clinical activity was limited with monotherapy or in combination with pembrolizumab (ORR 7% and 9.1%, respectively), whereas the chemotherapy arm achieved a higher ORR of 30.8%. In the phase 1/1b trial (NCT04306900), combination with budigalimab and chemotherapy achieved an ORR of 56.1% in gastric cancer and 33.3% in PDAC, with safety consistent with standard regimens. These findings suggest that CD39 inhibition is unlikely to be effective as monotherapy but may potentiate chemotherapy-based regimens.

IPH5201, a fully human anti-CD39 antibody, was evaluated in 57 patients with advanced solid tumours, most commonly pancreatic, NSCLC and colorectal cancers. The antibody was well tolerated with no dose-limiting toxicities, and pharmacodynamic analyses confirmed target engagement. Clinically, 40% of patients achieved stable disease as the best response, indicating disease-stabilising activity in heavily pretreated populations (Ref. 93).

SRF617 has been assessed in combination with etrumadenant (A2A/A2B antagonist) and zimberelimab (anti-PD-1) in metastatic castration-resistant prostate cancer (NCT05177770). Although the triplet regimen was generally manageable, no confirmed PSA50 (≥50% decline in prostate-specific antigen) or radiographic responses [complete response (CR) or partial response (PR)] were observed, and a high rate of severe adverse events (including fatigue, nausea, anaemia and appetite) was reported, underscoring the challenge of combining CD39 blockade with multi-agent regimens in heavily pretreated patients.

In addition, several additional agents, including JS019 (fully human anti-CD39 antibody, NCT05374226 and NCT05508373), ES014 (CD39/TGF-β bispecific, NCT05381935, NCT05717348 and NCT06543056), PUR001 (anti-CD39, NCT05234853) and ES002 (NCT05075564), have entered early-phase clinical evaluation but have not yet reported results.

Collectively, these early-phase trials demonstrate that CD39-targeted therapies have generally manageable safety profiles, particularly with TTX-030 and IPH5201, where no dose-limiting toxicities were observed. In contrast, SRF617-based triplet blockade was associated with substantial adverse events, underscoring the challenges of multi-agent regimens in heavily pretreated cohorts. Overall, CD39 inhibition appears feasible but remains experimental; randomised studies with rational combinations and biomarker-driven stratification will be essential to define its therapeutic value and long-term safety.

## Safety considerations in CD39-targeted immunotherapy

The safety profile of CD39-targeted therapies must be interpreted in light of the broad physiological role of CD39. Beyond its immunoregulatory function on regulatory and exhausted T cells, CD39 is constitutively expressed on vascular endothelial cells and platelets, where it hydrolyses ATP and ADP to maintain vascular homeostasis and suppress platelet aggregation. This dual biology creates a therapeutic paradox: systemic blockade may enhance antitumour immunity but could also destabilise vascular integrity or predispose to thrombo-inflammatory events.

Early clinical experience indicates that CD39 inhibition is generally manageable in certain settings. TTX-030 and IPH5201 showed no dose-limiting toxicities, with infusion-related reactions and fatigue being the most common treatment-related adverse events. By contrast, in the SRF617-based triplet study in metastatic castration-resistant prostate cancer, 43.75% of patients experienced serious adverse events, including cytokine release syndrome and infections, with a 25% mortality rate largely attributable to disease progression. Importantly, across all programmes, no consistent signal of thromboembolic complications has emerged to date, though larger cohorts and longer follow-up are required to evaluate chronic vascular safety.

## Challenges and perspectives in targeting CD39

Although CD39 has emerged as a promising target in cancer immunotherapy, several obstacles must be addressed before its full therapeutic potential can be realised. A major challenge lies in the complexity of the adenosinergic pathway. While CD39 inhibition reduces adenosine accumulation, adenosine also plays homeostatic roles in normal tissues, raising concerns about unwanted immune activation (Ref. 7). In addition, CD39 is widely expressed beyond immune cells, including on endothelial cells and platelets, creating risks of off-target effects such as vascular instability. A further limitation is the absence of reliable biomarkers to identify patients most likely to benefit from CD39 blockade (Ref. 7).

Another key translational challenge is the potential development of resistance mechanisms within the tumour microenvironment. Pathway redundancy represents a major barrier, as compensatory upregulation of CD73-mediated AMP hydrolysis may sustain adenosine production downstream of CD39 inhibition. Moreover, increased expression or signalling activity of adenosine receptors, particularly A2A and A2B receptors, may further preserve immunosuppressive signalling despite effective CD39 targeting (Ref. 30). In addition, sustained accumulation of extracellular ATP following CD39 blockade may paradoxically induce immune dysfunction through excessive activation of purinergic P2 receptors, including P2X7 (Ref. 94). Tumours may further adapt by reshaping the immune landscape towards alternative immunosuppressive cell populations, such as regulatory T cells or myeloid-derived suppressor cells, or by engaging parallel metabolic and cytokine-mediated pathways (Refs 94, 95). Together, these adaptive responses highlight the potential limitations of CD39 monotherapy and underscore the translational challenges associated with targeting CD39 alone.

Despite these challenges, several strategies offer promise to enhance therapeutic efficacy. Rational combination approaches, including dual inhibition of CD39 and CD73 or the integration of CD39 blockade with ICIs such as anti–PD-1 antibodies, may more effectively disrupt purinergic immunosuppression and restore antitumour immune responses, particularly in tumours refractory to monotherapies (Refs 15, 19, 74). In parallel, the development of more selective agents that preferentially target tumour-associated CD39 could mitigate safety concerns. Finally, the incorporation of predictive biomarkers, including spatial or functional readouts of purinergic signalling, together with continued investigation of compensatory pathways, will be critical for improving patient stratification and treatment durability.

## Conclusion

CD39 is now recognised as a central mediator of adenosine-driven immunosuppression and a compelling target for next-generation cancer immunotherapies. By hydrolysing extracellular ATP, CD39 enables tumour, stromal, and immune compartments to evade immune surveillance, and its inhibition represents a rational approach to restore antitumour immunity (Ref. 10). Preclinical studies and early-phase trials of monoclonal antibodies and small-molecule inhibitors have provided encouraging signals of safety and biological activity, particularly when combined with chemotherapy or immune checkpoint blockade. Nevertheless, important challenges remain. The redundancy of the adenosinergic pathway, together with the physiological expression of CD39 on endothelial cells and platelets, raises concerns regarding clinical efficacy and vascular safety. Furthermore, durable benefit and optimal treatment settings remain to be defined. Moving forward, refinement of inhibitor selectivity, development of spatial/functional biomarkers for stratification, rational combinations with established immunotherapies, and rigorous clinical evaluation will be essential to translate CD39-targeted strategies into effective treatments. Collectively, these efforts may unlock the therapeutic potential of CD39 inhibition as part of the next wave of cancer immunotherapy.
